# Health promotion interventions for African Americans delivered in U.S. barbershops and hair salons- a systematic review

**DOI:** 10.1186/s12889-021-11584-0

**Published:** 2021-08-16

**Authors:** Kelly N. B. Palmer, Patrick S. Rivers, Forest L. Melton, D. Jean McClelland, Jennifer Hatcher, David G. Marrero, Cynthia A. Thomson, David O. Garcia

**Affiliations:** 1 Department of Health Promotion Sciences, Mel and Enid Zuckerman College of Public Health, University of Arizona, 1295 N Martin Avenue, Tucson, AZ 85721-0202 USA; 2 Health Sciences Library, University of Arizona, 1295 N Martin Avenue, Tucson, AZ 85721 USA; 3 Division of Public Health Practice, Mel and Enid Zuckerman College of Public Health, 550 E. Van Buren Street, UA Phoenix Plaza Building 1, Phoenix, AZ 85006 USA

**Keywords:** African Americans, Chronic diseases, obesity, Cancer, Cardiovascular disease, Type 2 diabetes mellitus, Health promotion, Barbershops, Hair salons, Systematic review

## Abstract

**Background:**

African American adults suffer disproportionately from obesity-related chronic diseases, particularly at younger ages. In order to close the gap in these health disparities, efforts to develop and test culturally appropriate interventions are critical.

**Methods:**

A PRISMA-guided systematic review was conducted to identify and critically evaluate health promotion interventions for African Americans delivered in barbershops and hair salons. Subject headings and keywords used to search for synonyms of ‘barbershops,’ ‘hair salons,’ and ‘African Americans’ identified all relevant articles (from inception onwards) from six databases: Academic Search Ultimate, Cumulative Index of Nursing and Allied Health Literature (CINAHL), Embase, PsycINFO, PubMed, Web of Science (Science Citation Index and Social Sciences Citation Index). Experimental and quasi-experimental studies for adult (> 18 years) African Americans delivered in barbershops and hair salons that evaluated interventions focused on risk reduction/management of obesity-related chronic disease: cardiovascular disease, cancer, and type 2 diabetes were included. Analyses were conducted in 2020.

**Results:**

Fourteen studies met criteria for inclusion. Ten studies hosted interventions in a barbershop setting while four took place in hair salons. There was substantial variability among interventions and outcomes with cancer the most commonly studied disease state (*n* = 7; 50%), followed by hypertension (*n* = 5; 35.7%). Most reported outcomes were focused on behavior change (*n* = 10) with only four studies reporting clinical outcomes.

**Conclusions:**

Health promotion interventions delivered in barbershops/hair salons show promise for meeting cancer screening recommendations and managing hypertension in African Americans. More studies are needed that focus on diabetes and obesity and utilize the hair salon as a site for intervention delivery.

**Trial registration:**

PROSPERO CRD42020159050.

**Supplementary Information:**

The online version contains supplementary material available at 10.1186/s12889-021-11584-0.

## Background

African Americans, the second largest minority group, account for 13.4% of the United States (U.S.) population [1]. African Americans are disproportionately burdened by obesity and related chronic diseases such as heart disease, cancer, and type 2 diabetes resulting in higher rates of morbidity and mortality than non-Hispanic whites (NHW) [2]. African Americans have the second highest prevalence for obesity and diabetes (46.8 and 12.7%, respectively) of any racial/ethnic group [3]. African Americans have two times the risk of having stroke or dying from cardiovascular disease, and have 50% more risk of having hypertension than NHW [4]. African Americans are at higher risk of developing colorectal cancer and their mortality rates for multiple myeloma and stomach cancer are double that of NHW [5]. Moreover, prostate cancer mortality risk is two times as high for African American men as compared to NHW men; while African American women have higher risk of death from breast and cervical cancers than NHW [5]. Many social determinants of health (wage gaps, substandard education and healthcare, and unethical housing policies) are associated with health disparities among African Americans [6, 7]. Historically, African Americans have had mistrust in the medical and research community making them less likely to see a primary care doctor and participate in health promotion research [8–11]. Strategies to engage African Americans in health promotion programs must consider cultural appropriateness when designing and implementing effective health promotion interventions.

By working with community partners that deliver services to African Americans and trusted health care providers that are members of the African American community, interventionists can identify socioeconomic risk factors and barriers to healthcare utilization, facilitate coordination of care and resources, and implement evidence-based interventions to address health disparities. Engaging African Americans in health promotion interventions has been challenging likely in part due to a lack of consideration of the role culture plays in components such as intervention attendance and adherence. Typically, interventions are located in settings that have been perceived historically as inaccessible or excluding to African Americans further exacerbating health inequities. Toward this end, public health practitioners have turned to trusted community-based settings as sites for health promotion education and programming.

Health behavior researchers and programmers have utilized faith-based organizations to reach the African American community [12, 13]. The church has historically served as a source of refuge where members and the African American community at large can gather for non-religious purposes such as socialization and civic and political activities. As an integral part of the communities in which they reside, the church is often tasked with community outreach initiatives as well as economic development opportunities for local residents. Researchers and programmers can benefit from including core African American cultural constructs such as religiosity and social support/structure offered by the church in their interventions [14]. Furthermore, leveraging the social network by engaging leaders in the church that can reinforce participation or model the desired healthy behavior can be advantageous [15]. Integrating biblical texts and spiritual elements into the intervention increases program effectiveness [14]. For all the progress in reaching the African American community, there are limitations with church-placed and church-based interventions. Young African American adults and African American men are less likely than older African Americans and African American women to attend church services regularly [16]. Also, black churches have found themselves inundated with projects and competing interests making it difficult to prioritize health promotion programs [17].

Akin to the church, barbershops and hair salons are staples in the African American community. They impart important African American cultural constructs such as communalism and expressiveness [18]. Sociocultural influences of behavior can be explored and leveraged through the barbershop and hair salon. Barbershops and hair salons serve as sources of entrepreneurship; therefore, owners, barbers, and stylists alike are respected by members of the African American community. Because they are highly accessible, barbershops and hair salons have been involved in health promotion activities such as formative research, subject recruitment, and delivery/implementation of interventions [18–25]. Because African American men have traditionally been a difficult group to engage, barbershop health promotion has increased in popularity [26]. Oftentimes, African American men hang out for hours at the barbershop beyond their service visit. During this time one can network for a job, buy or sell products, advertise a business, watch movies or sports, discuss or get advice on personal and family affairs, and participate in other recreation (play board/video games, card, dominoes, etc.) [26].

Like their male counterparts, African American women maintain a high-level of engagement with the hair salon for many of the same reasons. Due to the unique and close relationship African American women have with their stylist, researchers can find opportunity in delivering interventions in hair salons and to a further extent by hair stylists [27–32]. Hair stylists are trusted by their clients and therefore serve as a confidante, a reliable source of information, and oftentimes as a close companion. This trust is in stark contrast to the mistrust of the medical system and research community common among African Americans. Because of this trust/mistrust, inaccessible quality healthcare, and lack of culturally appropriate interventions, African American woman are less likely to have a primary care provider [33]. However, it is more common for them to have a regular hair stylist illustrating the significance of routine hair care service [33]. Oftentimes hair care for African American women can require regular, lengthy visits to the salon thereby providing a captured audience suitable for health behavior interventions [34].

There is a paucity in the literature for systematic reviews that consider the role of the setting in engaging African Americans in health promotion. Among those, most have assessed cultural tailoring of evidence-based interventions (race concordance of interventionist, spirituality, etc.) [35–39]. A few have examined the role of churches, barbershops, and hair salons for recruitment of research participants into clinical trials [40, 41]. One synthesis of the literature explored barbershop and hair salon health promotion, but African Americans were not the primary population of interest [19]. Similarly, a 2015 qualitative systematic review described barber-led interventions targeted for African American men without inclusion of stylist-led interventions targeted for African American women [26]. This is the first systematic review of the effectiveness of barbershop and hair salon health promotion interventions for African Americans that elucidates the quality of evidence of these interventions. Characteristics of effective interventions addressing the leading obesity-related chronic disease (heart disease, cancer, and type 2 diabetes) health disparities for African Americans will be identified.

## Methods

### Literature search

This systematic review was conducted according to the guidelines set by the Preferred Reporting Items for Systematic Reviews and Meta-Analyses (PRISMA) statement [42]. The study was registered with the International Prospective Register of Systematic Reviews (PROSPERO) in 2020 (CRD42020159050). The detailed prespecified protocol has been previously published [43]. Seven databases (Academic Search Ultimate, Cumulative Index of Nursing and Allied Health Literature (CINAHL), Embase, PsychInfo, PubMed, Web of Science, and ProQuest Dissertations from inception to October 2019) were queried following comprehensive search strategies developed in consultation with a medical librarian (Additional file 1). Controlled vocabulary terms in databases (including MeSH and Emtree) and keywords were used in the search relevant to the target population (African Americans) and intervention component (delivery site- barbershops and/or hair salon) resulting in the following terms: “African American,” “Black American,” “African Ancestry,” “barber,” “barbering,” “beautician,” “beauty culture,” “cosmetologist,” “hair,” “hairdresser,” “hairstylist,” “stylist,” “beauty shop,” “beauty salon,” “hair salon,” and “salon.” The final search was conducted on October 08, 2019.

### Inclusion criteria/study selection

Studies were included if they met the following inclusion criteria:

1. Adult African Americans were the target population for the intervention.
2. The intervention was delivered in a U.S. barbershop or hair salon.
3. The study evaluated an intervention aimed at reducing risk factors or improving health outcomes of obesity and/or related chronic conditions (i.e. cardiovascular disease, cancer, and type 2 diabetes).

Only interventional study designs were included. Studies were excluded if participants were children/adolescents (aged < 18 years), the intervention took place outside of the U.S., or if the article was published in a language other than English.

### Identification of eligible articles

**Fig. 1:**
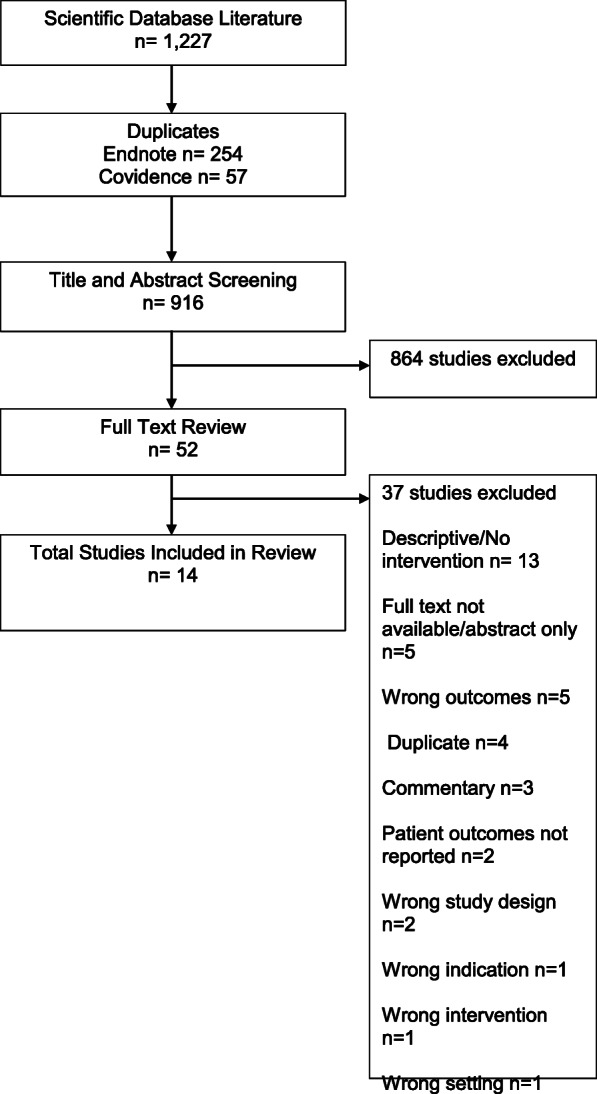
PRISMA flowchart

Figure 1 displays the screening and inclusion process depicted in a flow diagram. A search of the electronic databases yielded 1227 records by study author JM. After duplicates were removed, 973 records were uploaded to Covidence (Veritas Health Innovation, Melbourne Australia). Fifty-seven duplicates were removed, and 916 articles remained. Titles and abstracts were reviewed in triplicate by three of the study authors (KP, PR, and FM) resulting in 57 articles for full-text review. Study authors KP and PR independently reviewed the full text of each article against the inclusion/exclusion criteria. This resulted in 13 articles for inclusion in this review. One article reported on 2 studies, 2 articles are from the same study, but report different outcomes, and 2 articles are from the same study with one article reporting outcomes after extending the intervention [21, 29, 30, 44, 45].

### Data extraction and quality assessment

Data extraction was completed in duplicate by study authors KP and PR using a customized Research Electronic Data Capture (REDCap) database (Additional file 2) [46]. Data reports were reviewed for accuracy by FM. Data variables extracted from each article included: first author’s last name, year of publication, article title, sample size, age range or mean age, gender, socioeconomic status of participants, geographic location, disease focus, study design, study setting, intervention/control description, intervention duration, follow up time points, if a community-based participatory research approach was employed, interventionist, if culturally-sensitive strategies were implemented, if incentives were given, theoretical frameworks/models, barbershop/hair salon recruitment strategies, and study outcomes and results (noting significance). For studies where the barber/stylist was the interventionist, data on intervention training and strategies for intervention fidelity were also collected. Due to the heterogeneity of studies and outcomes, data were analyzed and synthesized for presentation narratively and in tables in 2020. Two authors (KP and PR) independently evaluated the quality of evidence using the Effective Public Health Practice Project Quality Assessment Tool (EPHPP) to increase inter-rater reliability and reduce risk of bias [47, 48]. Articles were given a global rating by each of the two reviewers of weak, moderate, or strong based on the six component ratings of selection bias, study design, confounders, blinding, data collection, and withdrawals/dropouts. The two reviewers discussed global ratings for each article and a final decision of both reviewers was recorded in a REDCap database (Additional file 3).

## Results

### Study characteristics

Because one article reported outcomes for two studies [21], 14 studies are included in the final review. Characteristics of the 14 studies are presented in Table 1. Studies were published between 2007 and 2019. Seven studies were randomized control trials (RCTs) (six cluster RCTs and 1 RCT), four were pretest-posttest (three 2- group and one 1-group), two were nonrandomized feasibility studies, and one (1 group) posttest only study. Sample sizes varied widely from 20 to 1297 participants. Mean age of study participants ranged from 37 to 57.4 years, but ranges were wide with participants aged 18 to 88. Socioeconomic status (SES) was reported by all but two studies. Participants in three studies were reported as having only a high school education or less, low-income, and/or mostly uninsured [20, 54, 55]. Studies were mostly conducted in large urban/metropolitan cities with only one in a rural area [20]. Seven interventions focused on outcomes related to cancer [27, 29, 50–53, 55], five on cardiovascular disease (i.e. blood pressure) [21, 44, 45, 49], one on type 2 diabetes [30], and one on obesity [20]. Barbershops accounted for the majority of study settings (*n* = 9) [21, 44, 45, 50–53, 55, 56], with four studies taking place in hair salons [20, 27, 29, 30]. Interventions taking place in barbershops targeted men (*n* = 9) [21, 44, 45, 50–53, 55, 56] while those in hair salons targeted women (*n* = 4) [20, 27, 29, 30].

**Table 1:** Study Characteristics

First Author, Year, Ref	Title	Sample Size	Mean Age/ Age Range	SES	Geographic Location	Study Design	Disease State/Focus	Setting
Hess, 2007 [21]	Barbershops as Hypertension Detection, Referral, and Follow-Up Centers for Black Men	*n* = 94	40–60	mostly insured or have access to public health care system	Dallas, Texas	Non-Randomized Feasibility	Cardiovascular Disease	Barbershop
Hess, 2007 [21]	Barbershops as Hypertension Detection, Referral, and Follow-Up Centers for Black Men	*n* = 321	40–60	mostly insured or have access to public health care system	Dallas, Texas	Non-Randomized Feasibility	Cardiovascular Disease	Barbershop
Wilson, 2008 [27]	Hair Salon Stylists as Breast Cancer Prevention Lay Health Advisors for African American and Afro-Caribbean Women	*n* = 1185	38	Not reported	Brooklyn, New York	Cluster Randomized Control Trial	Cancer	Hair Salon
Holt, 2010 [49]	Cancer Awareness in Alternative Settings: Lessons Learned and Evaluation of the Barbershop Men’s Health Project	*n* = 163	45+	Not reported	Birmingham, Alabama	2 group Pretest-Posttest	Cancer	Barbershop
Johnson, 2010 [20]	Beauty Salon Health Intervention Increases Fruit and Vegetable Consumption in African-American Women	*n* = 20	18–70	> 50% (11/20) High School Diploma	Rural South Carolina	2 group Pretest-Posttest	Obesity	Hair Salon
Luque, 2011 [50]	Barbershop communications on prostate cancer screening using barber health advisers	*n* = 40	53	mean education = 14 years, mean household income <$70 k, 78% privately insured	Tampa, Florida	1 group Posttest only	Cancer	Barbershop
Sadler, 2011 [29]	A Cluster Randomized Controlled Trial to Increase Breast Cancer Screening Among African American Women: The Black Cosmetologists Promoting Health Program	*n* = 984	40.6 20–88	mostly college educated (52% some college, 34% complete college)	San Diego, California	Cluster Randomized Control Trial	Cancer	Hair Salon
Victor, 2011 [27]	Effectiveness of a Barber-Based Intervention for Improving Hypertension Control in Black Men	*n* = 1297	Intervention: 49.5 Control: 51.2	85% middle income and insured	Dallas, Texas	Cluster Randomized Control Trial	Cardiovascular Disease	Barbershop
Odedina, 2014 [51]	Development and assessment of an evidence-based prostate cancer intervention programme for black men: the W.O.R.D. on prostate cancer video	*n* = 142	50–59	> 50%: <$20 k, H.S. diploma, had insurance, had PCP	Florida	1 group Pretest-Posttest	Cancer	Barbershop
Sadler, 2014 [30]	Lessons learned from The Black Cosmetologists Promoting Health Program: A randomized controlled trial testing a diabetes education program	*n* = 984	40.6 20–88	mostly college educated (52% some college, 34% complete college)	San Diego, California	Cluster Randomized Control Trial	Type 2 Diabetes	Hair Salon
Frencher, 2016 [52]	PEP Talk: Prostate Education Program, Cutting Through the Uncertainty of Prostate Cancer for Black Men Using Decision Support Instruments in Barbershops	*n* = 120	40+	majority income<$24 k; uninsured, college or more	South Los Angeles, California	2 group Pretest-Posttest	Cancer	Barbershop
Cole, 2017 [53]	Community-Based, Preclinical Patient Navigation for Colorectal Cancer Screening Among Older Black Men Recruited From Barbershops: The MISTER B Trial	*n* = 731	57.4 50+	mean annual income = $16,726, 1/3 < High School diploma, ~ 50% unemployed	New York, New York	Randomized Control Trial	Cancer	Barbershop
Victor, 2018 [45]	A Cluster-Randomized Trial of Blood- Pressure Reduction in Black Barbershops	*n* = 319	Intervention: 54.4 Control: 54.6 35–79	mostly college educated, have regular medical provider and insured	Los Angeles, California	Cluster Randomized Control Trial	Cardiovascular Disease	Barbershop
Victor, 2019 [46]	Sustainability of Blood Pressure Reduction in Black Barbershops	*n* = 319	I: 54.4 C: 54.6 35–79	mostly college educated, have regular medical provider and insured	Los Angeles, California	Cluster Randomized Control Trial	Cardiovascular Disease	Barbershop

### Interventions

Table 2 summarizes characteristics of the interventions. All studies evaluated barbershop/hair salon-based health promotion interventions aimed at reducing risk factors for or improving health outcomes of obesity-related chronic conditions in African Americans. Interventions were extremely heterogenous in mode of delivery, duration, and content. Most interventions were delivered in-person, two were delivered via media (video/DVD) [50, 52], and one was delivered via phone calls [55]. Barbers and stylists served as the interventionists in most cases. In two studies, when not serving as the primary interventionist, barbers and stylists supported client engagement with the interventionist, a medical professional/pharmacist [44, 45]. One study employed African American actors to portray barbers, barbershop clients, and doctors in a video-based intervention [52]. Two interventions were led by the researcher/research staff and one by trained counselors and community health workers [21, 50, 55]. Intervention duration varied from 25 min (video) to 14 months. The use of Social Cognitive Theory (SCT) was reported in three studies [21, 27]; two studies cited Health Belief Model [29, 30]; one study employed the Personal Integrative Model of Prostate Cancer and the Health Communication Process model [52], one study utilized peer learning [44], and one study adapted a model from the AIDS Community Demonstration Project [56]. Only five studies explicitly stated using a community-based participatory research (CBPR) approach [27, 29, 50, 51, 53].

**Table 2:** Intervention Characteristics

Author, Year	Setting	Intervention	Comparison	Interventionist	Duration/ Data Collection Time Points	Theoretical Framework/ Model	CBPR Approach	Recruitment Strategies	Culturally Adapted Strategies	Incentives
Hess, 2007 [21]	Barbershop	Staff delivered intervention- Physician referral for follow up with BP report card for ongoing feedback Role model stories depicting successful risk reduction strategies adopted by hypertensive African American men	Both groups received written results of the 3 BP screenings and standard recommendations for interval medical follow-up	Researcher/Research Staff	8 months/ Baseline and post-intervention	Social Cognitive Theory	Not reported	Not reported	Intervention delivered by African American research assistants and medical/premedical students supervised by an African American nurse	Barbers Customers
Hess, 2007 [21]	Barbershop	Barbers delivered the intervention- Blood Pressure report cards to be signed by provider and returned to barber	American Heart Association brochures titled High BP in African Americans	Barbers	14 months/ Post-intervention	Social Cognitive Theory	Not reported	Not reported	Not reported	Barbers Customers
Wilson, 2008 [27]	Hair Salon	Intervention designed to promote stylist’s skills and motivation to provide correct and consistent breast health info to female clients on an ongoing basis. Breast health recommendations included monthly breast self-exams, annual clinical breast exams, and routine mammography for women 40 + . Stylists to promote client skills, self-efficacy, and motivation for engaging in breast health behaviors Written materials for clients on where to get services for breast cancer detection and treatment	No-treatment control	Hair Stylists	3 months/ Baseline and 1–3 post-intervention	Social Cognitive Theory	Yes	List of salons from targeted neighborhoods generated via phone book listings and internet by zip codes. Randomly selected salons and contacted owners to assess willingness to participate in study.	No description	Hair Stylists
Holt, 2010 [51]	Barbershop	Health messages about CaP and CRC delivered by barbers to clients. Barbers help with strategies for informed decision making about screening supported by posters, print materials, and videos	Not reported	Barbers	3 months/ Baseline and post-intervention	Not reported	Yes	Barbershops recruited and trained by the community partnership	Community advisory panel developed intervention and recruited barbers	Customers
Johnson, 2010 [20]	Hair Salon	3 scripted motivational sessions during clients’ service appointments encouraging them to adopt healthy behaviors- 1) Role modeling 2) Motivation 3) Check-in and recognition Information packets- 4 pages of info on fruit/vegetable consumption, PA, and water consumption reviewed by dieticians Starter kits- Samples of fruits/vegetables and a bottle of water given at sessions 1 and 3	No treatment control at second salon	Hair Stylists	6 weeks/ Post-intervention	Not reported	Not reported	Stylists were screened to assess value of evidence-based health and any changes to the stylist’s personal health in the last 12 months.	Broad overall health changes instead of specific numerical goals with focus on efficacy. Materials reviewed by African American women before study	Not reported
Luque, 2011 [53]	Barbershop	CaP education materials developed by research team (brochure/poster, video, and Flipchart) tailored for African American men adapted from early detection/screening to informed decision-making for PCS guidelines. Plastic prostate model, barber talking points card, and community resources list	Not applicable	Barbers	one session during client visit to barbershop/ post-intervention	Not reported	Yes	Community health agency helped identify 2 barbershops. Snowball strategy from initial 2 barbershops resulting in 2 more barbershops. Clients- convenience sample of barbershops	Education materials tailored for African American men via learner verification and then piloted with African American men.	Not reported
Sadler, 2011 [29]	Hair Salon	Cosmetologists were to engage clients in conversation about adhering to BC screening guidelines for them, family, and friends, and importance of early detection (CBE and mammography) and treatment. A series of eight laminated “Mirror Challenges” were sequentially posted in a corner of the cosmetologists’ mirror. Relevant articles from lay newspapers and magazines trusted by the African American community were laminated and given to cosmetologists. A 3-ring binder of info was used as well. A soft plastic BC model to show how a BC lump felt and string of clay beads to depict various sizes of BC lumps given. BC posters with images of African American women throughout salon.	Diabetes education intervention identical to BC intervention in all ways but content	Hair Stylists	6 months/ baseline and 6 months	Health Belief Model	Yes	African American church members helped recruit cosmetologists and facilitate meeting with study leader. Clients recruited via African American research assistant or stylists.	Ancestral storytelling	Hair Stylists Customers
Victor, 2011 [49, 56]	Barbershop	Barbers offered repeated BP checks during haircuts, gave repeated personalized sex-specific health messages to promote physician follow up Posters with barbershop patrons modeling HTN treatment behaviors and testimonials Patrons with elevated BP recommended to follow up with a physician (or study nurse) Patrons with elevated BP received referral cards to give physicians for feedback and to document patron-physician interaction	Standard HTN education pamphlets from the AHA written for a broad audience of black men and women	Barbers	10 months/ Baseline and 10 months	Adapted from the AIDS Community Demonstration Projects that mobilized community peers to deliver intervention messages (specific action items) with role model stories and made medical equipment available in the daily environment	Not-reported	Barbershops selected to represent 4 geographic areas > 95% black male clientele > 10 years in business > 3 barbers	Not-reported	Barbers Customers
Odedina, 2014 [52]	Barbershop	A prostate cancer education video “Working through Outreach to Reduce Disparity (W.O.R.D.) on Prostate Cancer” Focuses on explaining the risk factors for CaP, how to reduce the risk for CaP, and informed decision making about CaP screening. Barbershop conversation teaches main character importance of CaP prevention (CaP survivor shares his story). As a result, he decides to follow up with doctor.	Not applicable	African American actors portraying barbers, clients, ministers, and doctors	25 min/ Baseline and post-intervention	Personal Integrative Model of Prostate Cancer Disparity (PIPCaD) model Health Communication Process Model	Not reported	Not applicable	Using African American actors to model desired behaviors for target population (African American men) Video setting in a barbershop	Customers
Sadler, 2014 [30]	Hair Salon	Diabetes education intervention to increase diabetes knowledge, change diabetes attitudes, and increase diabetes screening behaviors among African American women. Article references Sadler 2011 with details of BC intervention that is comparable to diabetes intervention with only difference being content.	BC education intervention identical to diabetes intervention in all ways but content	Hair Stylists	6 months/ baseline and 6 months	Health Belief Model	Not reported	African American church members helped recruit cosmetologists and facilitate meeting with study leader. Clients recruited via African American research assistant or stylists.	Ancestral storytelling	Hair stylists
Frencher, 2016 [50]	Barbershop	2 Decision Support Instruments in DVD format: VCU- culturally tailored to African American men FIMDM- general audience Both present treatment options for CaP	DSI DVD designed for general audience	Researcher/Research Staff	One-time intervention, 30 min/ 3 months post-intervention	Not reported	Yes	Recruited from Black Barbershop Health Outreach Program (BBHOP) and other non-BBHOP barbershops. Recruitment was scripted and letters of support and consent for research were obtained from owners.	VCU’s DSI DVD tailored to African American men using focus group data from African American men to develop the decision tool. The cast in the video are mostly African American	Barbers Customers
Cole, 2017 [55]	Barbershop	3 arms (PN, MINT, PLUS); cross randomized PN: Patient navigation for CRC screening. 2+ phone calls: 1) education 2) screening readiness assessment & barriers. PN encourage colonoscopy appt. Within 2 weeks. Or FIT if preferred.	MINT: motivational interviewing and goal setting, 4 sessions PLUS: PN + MINT All: Printed education materials from American Cancer Society and NHLBI	CHWs/Trained Counselors	6 months/ 2 weeks and 6 months	Not reported	Not reported	Barbershops were identified by study staff from densely populated African American neighborhoods. Participants (customers and local residents) recruited during screening event at barbershop.	Not reported	Not reported
Victor, 2018 [44]	Barbershop	Barbers measured BP and encouraged follow up with pharmacist Pharmacists met regularly with participants in barbershops, prescribed meds, measured BP, encouraged lifestyle changes, and monitored plasma electrolyte levels Pharmacists followed up with participants’ physician (via progress notes) Pharmacists interviewed participants to generate peer-experience stories (posted on shop walls), reviewed blood-pressure trends, and gave participants $25 per pharmacist visit to offset the costs of generic drugs and transportation to pharmacies. 2 BP screening results with follow up recommendations and identification cards, follow up calls at 3mos, culturally specific health sessions, and vouchers for haircuts	Active control approach (in which barbers encouraged lifestyle modification and doctor appointment)	Medical Professionals-Pharmacists	6 months/ Baseline and 6 months	Peer learning	Not reported	Not reported	No description	Customers
Victor, 2019 [45]	Barbershop	Barbers measured BP and encouraged follow up with pharmacist Pharmacists met regularly with participants in barbershops, prescribed meds, measured BP, encouraged lifestyle changes, and monitored plasma electrolyte levels Pharmacists followed up with participants’ physician (via progress notes) Pharmacists interviewed participants to generate peer-experience stories (posted on shop walls), reviewed BP trends, and gave participants $25 per pharmacist visit to offset the costs of generic drugs and transportation to pharmacies. 2 BP screening results with follow up recommendations and identification cards, follow up calls at 3mos and 9mos, culturally specific health sessions, and vouchers for haircuts	Instruction about BP and lifestyle modification	Medical professionals-Pharmacists	12 months/ baseline, 6 months, and 12 months	Not reported	Not reported	Not reported	No description	Customers

*BP* Blood Pressure, *CaP* Prostate Cancer, *CRC* Colorectal Cancer, *PA* Physical Activity, *PCS* Prostate Cancer Screening, *BC* Breast Cancer, *CBE* Clinical Breast Examination, *AHA* American Heart Association, *HTN* Hypertension, *VCU* Virginia Commonwealth University, *DSI* Decision Support Instrument, *FIMDM* Informed Medical Decisions Foundation, *CHW* Community Health Worker

Barbershop/hair salon recruitment strategies were described for most of the studies. Five of the studies employed community agencies or existing community partnerships to recruit sites [29, 30, 50, 51, 53]. Three studies targeted certain geographical areas [27, 55, 56]. For two studies, hair stylists were assessed for fit with the mission of the project [20, 30]. And one study reported specific criteria for selection of barbershops [56]. Aside from using the barbershop or hair salon as the primary site for interventions, other culturally adapted strategies used were tailoring materials (print/media) for African Americans and in some cases specifically by gender. Materials were either developed or tested by the target audience prior to use in the studies. Other tactics were ensuring interventionists and/or data collectors were African American. One study incorporated ancestral storytelling, a traditional African communication model, as a mechanism for the hair stylists to deliver the intervention message to their clients and subsequently to their clients’ family and friends [30]. The majority of studies provided incentives to barbers/stylists and/or customers. Intervention content focused on the following topics: cancer (screening, prevention, treatment, medical provider engagement, access, risk factors, general knowledge), cardiovascular disease (blood pressure (BP)/hypertension (HTN) treatment, medical provider engagement, access), diabetes (screening, medical provider engagement, access, risk factors, general knowledge), obesity (physical activity, diet, water consumption), skill building, and self-efficacy to engage in the intended health behavior.

For interventions delivered by barbers or hair stylists (*n* = 8), details about intervention training and fidelity strategies are provided in Table 3. Trainings were in-person and facilitated by the researcher/research staff and when appropriate medical or content professionals. Written materials (handbooks, brochures, scripts, etc.) were used to supplement trainings as well as ongoing/refresher trainings. Intervention fidelity strategies included on-site monitoring by research staff or a community research partner, regular quality assurance review of the data being collected, and researcher accessibility to the barbers/stylists for continued support. One study did not monitor fidelity throughout the intervention, but did post-study surveys and interviews with study participants to evaluate barber intervention delivery [56].

**Table 3:** Barber/Stylist Led Interventions

Author, Year	Interventionist/Setting	Intervention Training	Intervention Fidelity Strategies
Hess, 2007 [21]	Barbers/Barbershop	Not reported	Research staff regularly checked the validity of the encounter form data against data stored in the electronic monitors and intermittently observed customer flow to validate the barbers’ counts of adult and child business.
Wilson, 2008 [27]	Hair Stylists/Hair Salon	Stylists were trained to conduct tailored and culturally sensitive counseling that would encourage clients to engage in breast health behaviors 2, two-hour workshops, a reference handbook, and ongoing support and technical assistance by research staff. Stylist training was implemented in waves, based on planned initiation of intervention activities in that salon	Program staff made frequent visits to salons to support stylists in their promotion of message delivery throughout the time during which the program was administered.
Holt, 2010 [49]	Barbers/Barbershop	Barbers trained by community advisory panel. One day of training education training modules and barbers given strategies for helping their clients make informed decisions about screening	Did not collect/not report.
Johnson, 2010 [20]	Hair Stylists/Hair Salon	Stylists were trained by research team. Motivational sessions using a script as a guide with practice and feedback from research team member.	Weekly check-ins.
Luque, 2011 [50]	Barbers/Barbershop	10 contact hours of training (didactic, interactive group, and team building) on administering materials by research team, health agency partners, and local urologist at agency’s facilities and in barbershops.	Health agency partner monitored barbers via shop visits, attended project meetings, and facilitated focus group work with barbers for post-intervention evaluation.
Sadler, 2011 [29]	Hair Stylists/Hair Salon	Cosmetologists received ~ 4 h of 1-on-1 training with the Principal Investigator and an additional 4 h of reading materials that reviewed and summarized the Principal Investigator’s training. The reading materials resources: National Cancer Institute, American Cancer Society, and Susan G. Komen-for-the-Cure Foundation. Cosmetologists also received individual training from an African American ancestral storyteller to enhance their ability to pass along their health promotion messages orally. Every two weeks, the cosmetologists were given hands-on training materials and shown ways the materials could be used to facilitate discussions with their clients to keep the screening message updated with fresh information	Principal Investigator made unannounced visits to salons every 2 weeks during the first 3 months and then monthly thereafter to restock and bring new materials (for consistency), offer training, and answer questions. Principal Investigator was accessible to cosmetologists at all times.
Victor, 2011 [27]	Barbers/Barbershop	Not reported	Participant follow up survey and interview data on intervention delivery by barbers.
Sadler, 2014 [30]	Hair Stylists/Hair Salon	IRB consent training. Stylists received 1-on-1 training with the Principal Investigator and reading materials. Stylists also received individual training from an African American ancestral storyteller to enhance their ability to pass along their health promotion messages orally. The stylists were given ongoing training from the Principal Investigator and participated in biannual luncheon trainings. Screening message updated with fresh information.	Principal Investigator and research team made unannounced visits to salons. Principal Investigator was accessible via cell phone to stylists at all times.

### Outcomes

Primary, secondary, and feasibility outcomes data are presented in Table 4. There was significant heterogeneity in outcomes with most primary outcomes being behavioral and four studies reporting clinical outcomes related to BP/ HTN [21, 44, 45, 56]. Behavioral outcomes included clinic-based screening (completion/intent), home-based screening (self-exam), provider follow up (treatment, general conversation), physical activity (quantity), and diet (servings of fruit/vegetables and water consumption). Six studies reported significant between-group differences [20, 21, 44, 45, 55, 56] while two reported significant with-in group differences [52, 53]. All studies that reported changes in HTN treatment had significant findings. Two articles where each intervention served as the comparison for the other, reported significant outcomes related to cancer screening for both groups, but non-significant results for the diabetes screening (between-group and intervention). The interventions were identical in every aspect except content (specific to disease state) [29, 30].

**Table 4:** Outcomes

Author, Year	Setting	Primary Outcomes	Primary Results	Secondary Outcomes	Secondary Results (Significant)	Feasibility Outcomes	Feasibility Results
Hess, 2007 [21]	Barbershop	Change in BP Changes in HTN Treatment rate (percentage of hypertensive subjects receiving prescription BP medication) HTN control rate	I: BP fell 16 +/− 3/9 +/− 2 mmHg (systolic: 149.1 +/− 2.2 to 133.4 +/− 2.2 mmHg; diastolic: 87.4 +/− 2.6to 78.82.6 mmHg) C: Unchanged (systolic: 146.4 +/− 2.4 to 146.7 +/− 2.4 mmHg; diastolic: 87.9 +/− 2.2 to 88.0 +/− 2.2 mmHg) Intervention effectremained significant (*P* < 0.0001) after adjustment for age and body mass index I: HTN treatment increased from 47 to 92% (*P* < 0.001) C: Unchanged I: HTN control increased from 19 to 58% (P < 0.001) C: Unchanged			Implementation	high percentage of haircuts accompanied by a BP recording, as well as BP readings interpreted correctly.
Hess, 2007 [21]	Barbershop	Proportion of haircuts in which the barber recorded a BP	81% haircuts barber recorded a BP	HTN control rate	HTN control rate increased progressively with increasing levels of intervention exposure: 20+/− 10.7% to 51+/− 9% (*p* = 0.01) Association between intervention exposure and HTN control remained significant after controlling for insurance status (p = 0.01)	Implementation	high percentage of haircuts accompanied by a BP recording BP readings interpreted correctly. Barbers correctly staged 92% of BPs
Wilson, 2008 [27]	Hair Salon	Self-breast exam (BSE) completion Clinical breast exam (CBE) completion CBE intention (12 months) Mammogram completion Mammogram intention (12 months)	BSE completion: AOR 1.60 (95% CI: 1.2–2.13) CBE completion: AOR 1.20 (95% CI: 0.94–1.52) CBE intention: AOR 1.87 (95% CI: 1.11–3.13) Mammogram completion: AOR 1.21 (95% CI: 0.84–1.76) Mammogram intention: AOR 1.34 (95% CI: 0.9–1.2)			Implementation- degree of execution	37% intervention vs. 10% control reported exposure to breast health messages
Holt, 2010 [51]	Barbershop	CaP screening/intent to screen (PSA/DRE) CRC screening/intent to screen (FOBT/FS/CS)	Possible increases in self-reported PSA test and prep for PSA and DRE. I: constantly greater increase in awareness, screening, and prep for FS	CaP knowledge CRC knowledge CRC screening perceived barriers and benefits	Results not significant	Not reported	Not reported
Johnson, 2010 [20]	Hair Salon	Increase in fruit and vegetable consumption Increase in physical activity Increase in water consumption	Fruit and vegetable intake increased from pre-posttest for the treatment group No increase in physical activity No increase in water consumption			Not reported	Not reported
Luque, 2011 [53]	Barbershop	Likelihood of discussing CaP with healthcare provider (4-point Likert scale (very unlikely to very likely)) CaP knowledge (5 pt. Likert scale (low to high))	Somewhat likely to very likely Increased from 75 to 85% *p* < .001 78% reported increase in knowledge	Feelings of worry about CaP (4 pt. Likert not worried to very worried) Projected PCS modality intention (PSA, DRE, or both)	Somewhat worried to very worried increased from 35 to 45%. p < .001 85%- Both (PSA & DRE)	Satisfaction with the intervention Intention to continue the intervention Expansion and implementation	Participants reported that the materials were easy to understand, had an attractive color scheme, and featured familiar faces printed on the materials. All barbershop clients surveyed reported positively on the contents of the brochure and poster 53% had discussed CaP at least two times with their barber in the last month
Sadler, 2011 [29]	Hair Salon	Adherence to Mammography screening guidelines	ITT between groups at follow up not significant ITT for mammography completers in both groups significantly (*p* < .05) higher at follow up. Adjusting for age (40+) as covariate yielded adherence to screening OR 2.0 (95% CI: 1.03–3.85) times higher for I vs C	Clinical breast exam adherence Participants’ awareness and perceptions of their vulnerability for breast cancer	ITT for perception of seriousness of BC as health threat reduced significantly (p < .05) in both groups, but greater reduction in diabetes arm. OR of listing BC as threat 1.8 times higher in BC arm (95% CI: 1.0–3.1).	Practicality Implementation- degree of execution	57% of the women reported that health education materials were displayed in their salon 57% participants reported that the cosmetologists in their salon were offering health information to their clients 80% of the women felt cosmetologists could effectively carry out intervention
Victor, 2011 [49, 56]	Barbershop	Change in HTN control rates (BP measurements and prescription labels) Patron-physician follow up interaction (signed referral card)	Greater HTN control in I vs C Intervention effect: Absolute group difference- 8.8% (95% CI: 0.8–16.9; Unadjusted: p = .04 Adjusted *p* = .03) Intervention effect: ITT- 7.8% (95% CI: 0.4–15.3; *p* = .04)	Barbershop-level changes in HTN treatment rates HTN awareness BP levels	Results not significant	Satisfaction with the intervention Intention to continue the intervention Practicality Implementation and Penetration	83% patrons heard a model story during every one or half their haircuts from barber 77% patrons received BP measurement from barber 51% patrons with elevated BP received counseling/physician referral from barber 98% patrons and all 29 barbers would like the intervention to continue Cost analysis- Cost effectiveness- cost-neutral for health care system would be $50/patron
Odedina, 2014 [52]	Barbershop	CaP screening CaP knowledge Decisional conflict	CaP Screening intention: 12.78 (2.48) to 13.37 (2.13) *p* = .0001 CaP knowledge: 63.60 (22.20) to 74.00 (16.80) *p* = 0.0021	Intervention effects	Completion of PN Intervention was significantly associated with study completion and CRC screening	Satisfaction with the intervention Limited Efficacy	> 90% of the participants indicated that they were satisfied with the video The mean satisfaction rating was 13.67 on a scale ranging from 3 to 15, indicating a highly satisfactory rating for the video > 75% of the participants indicated that the video: 1) was useful, 2) was understood, 3) not embarrassing, 4) was not too long, 5) not difficult, 6) was relevant, 7) got their attention, 8) has potential to increase CaP knowledge for African American men, and 9) was credible
Sadler, 2014 [30]	Hair Salon	Self-reported diabetes screening test in the past year, annual physical exam, and annual eye exam	There were no significant differences in rates of diabetes screening, routine annual screening, and eye exams from baseline to follow-up and between the two arms at follow-up	Knowledge and attitudes about diabetes	Both groups increased significantly from baseline in their overall diabetes knowledge: diabetes arm (M = 4.47; SD = 1.67) and breast cancer arm (M = 4.61; SD = 1.54), *P* < 0.05	Practicality Limited Efficacy Implementation- degree of execution	75% reported attending salon where health education was being offered. 65% reported cosmetologist made health info available 41% shared info w with family and friends 92% feel cosmetologist could effectively deliver diabetes information
Frencher, 2016 [50]	Barbershop	CaP screening via PSA test	*n* = 58 completed PSA testing (48%)	CaP knowledge and intention	Changes in knowledge and intention- all significant Intention to screen- increased from 57 to 73% Overall- no between group differences	Not reported	Not reported
Cole, 2017 [55]	Barbershop	CRC screening completion (self-report)	ITT; Mixed-effects regression analysis PN: 17.5% completion; MINT: 8.4%; PLUS: 17.8% PN: AOR = 2.28; 95% CI = 1.38, 4.34; PLUS: AOR = 2.44; 95% CI = 1.38, 4.34 2xs more likely for CRC screening completion (PN and PLUS) intraclass correlation coefficient = 0.039			Not reported	Not reported
Victor, 2018 [44]	Barbershop	Changes/reduction in systolic blood pressure	I: 27.0 mmHg reduction in SBP C: 9.3 mmHg Mean reduction in SBP 21.6 mmHg > for I than C (95% CI: 14.7, 28.4); p < .001 ITT Intervention effect: 21.0 mmHg > for I than C (95% CI: 14.0, 28.0); *p* < .001	Changes in DBP Rates of meeting BP goals Numbers of hypertensive meds Adverse drug reactions Self-rated health Patient engagement	Mean reduction in DBP 14.9 mmHg > in I vs C (95% CI, 10.3 to 19.6; P < 0.001) I: higher % of meeting BP goals I: Increases in use of antihypertensive meds: 55–100%; C: 53–63% (p < .001)	Limited Efficacy Implementation- degree of execution	7 in-person pharmacist visits and 4 follow up calls per participant 6 calls/messages to pharmacist per participant 4 BP Checks per participant by barber 4 health lessons per participant by barber
Victor, 2019 [45]	Barbershop	Change in SBP	I: mean reduction = 28.6 mmHg C: mean reduction = 7.2 mmHg Mean SBP reduction 20.8 mmHg > I vs C (95% CI: 13.9, 27.7; p < 0.0001) ITT intervention effect: 20.6 mmHg reduction (95% CI: 13.8, 27.3; *p* < 0.0001)	Changes in DBP Rates of meeting BP goals Numbers of hypertensive meds Adverse drug reactions Self-rated health Patient engagement	Mean DBP reduction 14.5 mmHg > I vs C (95% CI, 9.5–19.5 mmHg; P < 0.0001) I: higher % of meeting BP goals (68% vs 11%; *p* = 0.0177) I: Increase in use of antihypertensive meds: 57 to 100% C: 53 to 65% No treatment-related adverse events/deaths I: Greater increase in self-rated health and patient engagement scores	Limited Efficacy Implementation- degree of execution	11 in-person pharmacist visits (0-6 months = 4;7-12 months = 4) 4 BP checks per participant by barber 4 health lessons per participant by barber

*BP* Blood Pressure, *SBP* Systolic Blood Pressure, *DBP* Diastolic Blood Pressure, *I* Intervention, *C* Control, *CaP* Prostate Cancer, *CRC* Colorectal Cancer, *PA* Physical Activity, *PCS* Prostate Cancer Screening, *PSA* Prostate Specific Antigen, *DRE* Digital Rectal Examination, *FOBT* Fecal Occult Blood Test, *FS* Flexible Sigmoidoscopy, *CS* Colonoscopy, *BC* Breast Cancer, *CBE* Clinical Breast Examination, *BSE* Breast Self-Examination, *HTN* Hypertension, *ITT* Intention to Treat

Feasibility outcomes included as an exploratory focus for this review were not always explicitly stated, but assessed the following areas: acceptability (satisfaction, intent to continue use), practicality (quality of implementation, effects on target audience, ability to carry out, cost analysis), integration, limited efficacy (effect size, intended effects on intermediate variables), and implementation (degree of execution, success or failure of execution). Implementation was the most assessed (*n* = 7) followed by acceptability (*n* = 5), practicality (*n* = 3), and limited efficacy (n = 3). Overall, studies reported favorable feasibility outcomes noting barbers/stylists’ ability to deliver, barber/stylists’ degree of executing the interventions, and clients’ satisfaction with interventions. In one study, 98% of participants and all of the barbers expressed a desire to continue with the intervention [56]. One study performed a cost-analysis for a barber delivered hypertension intervention. In the cost-effectiveness model, the intervention was cost-neutral with the intervention costing ~$50 per barbershop patron [56].

### Quality of evidence

Global evidence quality ratings for each study appear in Table 5. Guided by the EPHPP evaluation process, studies were rated on the following six components: selection bias, study design, confounders, blinding, data collection methods, and withdrawals/dropouts. The global rating for each study was determined based on the total number of component “weak” ratings. One study was rated as “strong,” [56] two rated as “moderate,” [44, 45] and eleven rated as “weak.” [20, 21, 27, 29, 30, 50–53, 55] Many of the studies that had a global rating of “weak” had non-RCT study designs resulting in a “moderate” or “weak” study design rating and components that did not apply/could not be rated accordingly. Because most participants were self-referred, many studies rated “weak” on selection bias. Oftentimes, studies did not report on blinding or on validation/reliability of data collection instruments and therefore received component ratings of “weak.” Most studies controlled for confounders during analysis yielding “strong” component ratings.

**Table 5:** Quality of Evidence

Author, Year	Study Design	EPHPP Global Quality Assessment Rating
Hess, 2007 [21]	Non-Randomized Feasibility	Weak
Hess, 2007 [21]	Non-Randomized Feasibility	Weak
Wilson, 2008 [27]	Cluster Randomized Control Trial	Weak
Holt, 2010 [51]	2 group Pretest-Posttest	Weak
Johnson, 2010 [20]	2 group Pretest-Posttest	Weak
Luque, 2011 [53]	1 group Posttest only	Weak
Sadler, 2011 [29]	Cluster Randomized Control Trial	Weak
Victor, 2011 [49, 56]	Cluster Randomized Control Trial	Strong
Odedina, 2014 [52]	1 group Pretest-Posttest	Weak
Sadler, 2014 [30]	Cluster Randomized Control Trial	Weak
Frencher, 2016 [50]	2 group Pretest-Posttest	Weak
Cole, 2017 [55]	Randomized Control Trial	Weak
Victor, 2018 [44]	Cluster Randomized Control Trial	Moderate
Victor, 2019 [45]	Cluster Randomized Control Trial	Moderate

## Discussion

With the disproportionate rates of obesity-related chronic diseases in the African American community, there is an imperative need to better elucidate strategies for engagement in health promotion interventions. Due to historically unethical medical and research practices in the U.S., African Americans have a longstanding history of mistrust of the medical and research community resulting in low participation and engagement, furthering the gap in health [8, 57, 58]. To remedy this, the use of culturally “safe” spaces such as churches, barbershops, and hair salons for recruitment and engagement of African Americans into research studies and lifestyle/behavioral interventions have become increasingly popular [59–61]. To this end, designing interventions to be delivered in trusted, culturally significant settings are advantageous. Barbershops and hair salons are highly accessed, cultural staples in the African American community perfectly situated to tackle the health disparities that plague this community.

This is the first review to synthesize the effectiveness and feasibility of obesity-related chronic disease interventions targeted for African Americans delivered in barbershops and hair salons. Eight of the fourteen studies included in this review reported significant results for clinical and/or behavioral outcomes suggesting that interventions delivered in barbershops and hair salons may be effective for reducing risk factors for or improving health outcomes of obesity-related chronic conditions in African Americans. Of these studies, half used an RCT design, the most rigorous methodology for establishing effectiveness. However, only one of these studies received a “strong” global quality assessment rating, while two received a rating of “moderate” and one received a rating of “weak” due to deficiencies in blinding and selection bias, data collection methods and reporting of withdrawals/dropouts, respectively. This coupled with the variability of duration for interventions point to the need for more efficacious research with considerations for the nuances of community-based study designs.

Among the research with significant results, the outcomes are split evenly between clinical and behavioral. Clinical interventions focused on changes in blood pressure and HTN management while behavioral interventions that can support clinical outcomes included cancer (prostate and colorectal) screening. Furthermore, half of these interventions were delivered or supported by the barbers/hair stylists. Considered together, these details suggest that barbershop/hair salon-based interventions can have a valuable direct or indirect impact in health promotion research. Most studies evaluated feasibility elements, but those were not among primary outcomes nor did any studies compare intervention components such as setting (i.e. barbershop versus church/clinic/other community site) or interventionist (i.e. barber versus clinician/community health worker/researcher). One study where the researcher was the interventionist was replicated by the study team using the barber as the interventionist, but outcomes reported differed [21]. Future research would also benefit from examining the association of racial and gender congruence between the barber/stylist interventionist and clients and the desired outcomes. More research is needed to disentangle which components of the interventions are influencing outcomes.

### Limitations

There are some limitations of this systematic review. The heterogeneity of the studies (study designs, sample sizes, intervention characteristics, and outcomes) made it difficult to compare the effectiveness of intervention strategies. This was due in part to the inclusion of multiple disease states, however, there were small number of studies identified through a comprehensive search strategy. However, limiting the search to one disease state or outcome would have further restricted the number of relevant articles for inclusion. Smaller, non-RCT, short-term studies of moderate and weak quality did not support the evidence for or against the efficacy of barbershop/salon-based interventions. Self-reported data could have overpredicted effectiveness of interventions. Another limitation is that seven of the studies were conducted by the same three lead authors (three by one, two by one, and two by one) indicating potential publication bias. Finally, generalizability of the studies’ findings is questionable given most studies were conducted in large, urban cities with participants of higher socioeconomic status.

## Conclusions

Health promotion interventions delivered in barbershops and hair salons for African Americans appear to be modestly effective for reducing risk and improving health outcomes for obesity-related chronic diseases. Overall, the literature in this area is limited and varies in foci. The extent to which the barber/stylist is utilized warrants further investigation. Objective measurements could enhance results. While barbershops have been shown to be effective locations for recruitment of African American men, who have been the target audience for such interventions due to the difficulty with recruiting and engagement in health promotion interventions, research in hair salons with African American women deserves more attention. Moreover, interventions that address complex, layered behavior change associated with obesity and diabetes are needed while balancing the appropriateness of desired outcomes (behavioral versus clinical). While all community-based research can be involved and complicated, it can be gleaned from this literature that barbershops/hair salon-based interventions are feasible. The barbershop/hair salon and to a further extent, the barber and hair stylist, can serve to support the implementation of existing evidence-based interventions, possibly in partnership with the health care system, to address obesity and chronic disease health inequities for African Americans.

## Supplementary Information

**Additional file 1.** Search strategy.

**Additional file 2.** Data extraction form.

**Additional file 3.** Quality assessment form.

## Data Availability

The datasets used for the current study are available from the corresponding author on reasonable request.

## Abbreviations

BP: Blood pressure
CBPR: Community-based participatory research
EPHPP: Effective Public Health Practice Project Quality Assessment Tool
HTN: Hypertension
NHW: Non-Hispanic whites
PRISMA: Preferred Reporting Items for Systematic Reviews and Meta-Analyses
PROSPERO: International Prospective Register of Systematic Reviews
RCT: Randomized control trial
REDCap: Research Electronic Data Capture
SCT: Social cognitive theory
SES: Socioeconomic status
U.S.: United States
