# Supplementary material for: Health promotion interventions for African Americans delivered in U.S. barbershops and hair salons- a systematic review
Source: BMC Public Health. 2021 Aug 16;21:1553. doi: 10.1186/s12889-021-11584-0 (PMC8365990; doi:10.1186/s12889-021-11584-0)
Supplement: Supplementary file 1 — Additional file 1. Search strategy. [file 12889_2021_11584_MOESM1_ESM.docx]

Additional File 1. Search Strategy

| PubMed- October 2019 |
| --- |
| **African American or African Ancestry** |
| African American OR Black American OR African ancestry OR African continental ancestry group[MH] |
| **Hair Salons / Barbershops** |
| (barber NOT barber[au]) OR beautician OR hairdresser OR hairstylist OR beauty salon OR hair salon OR salon OR salons OR stylist OR stylists OR "Barbering"[Mesh] OR "hair"[mesh] OR "beauty culture"[mesh] OR "beauty shop" |
| **Combination** |
| (African American OR Black American OR African ancestry OR African continental ancestry group[MH]) AND ((barber NOT barber[au]) OR beautician OR hairdresser OR hairstylist OR beauty salon OR hair salon OR salon OR salons OR stylist OR stylists OR "Barbering"[Mesh] OR "hair"[mesh] OR "beauty culture"[mesh] OR "beauty shop") |
