# Supplementary material for: Health promotion interventions for African Americans delivered in U.S. barbershops and hair salons- a systematic review
Source: BMC Public Health. 2021 Aug 16;21:1553. doi: 10.1186/s12889-021-11584-0 (PMC8365990; doi:10.1186/s12889-021-11584-0)
Supplement: Supplementary file 2 — Additional file 2. Data extraction form. [file 12889_2021_11584_MOESM2_ESM.pdf]

Article Data Extraction

Article ID	________________ (enter ID # from Covidence)
Title	________________
First Author	________________
Year	________________
Sample Size	________________
Age range or mean age	________________
Socioeconomic Status	________________
Participants' Disease Status or Risk Factors	________________ (Did the participants have: Hypertension, Prediabetes, etc)
Geographic location	________________ (city, region, etc)
Disease state/focus	☐ Cardiovascular Disease<br>☐ Cancer<br>☐ Type 2 Diabetes<br>☐ Obesity
Study Design	◯ Randomized Control Trial<br>◯ Cluster Randomized Control Trial<br>◯ Posttest only<br>◯ Pretest-Posttest<br>◯ Other
Study Setting	◯ Barbershop<br>◯ Hair Salon<br>◯ Both
Intervention	________________ (Briefly describe)
Intervention Duration	________________ (reporting in weeks preferred)

Intervention follow up time points	__________________
CBPR approach?	◯ Yes<br>◯ No<br>◯ Not reported
Interventionist	☐ Barber/stylist<br>☐ Researcher/research staff<br>☐ Medical Professional<br>☐ Other<br>☐ Not reported
Other	__________________
Were culturally-sensitive strategies implemented?	◯ Yes<br>◯ No<br>◯ Not reported
Describe	__________________
Incentives	☐ Barber/Sylist<br>☐ Customers<br>☐ Both<br>☐ Not reported
Theoretical Frameworks/Models	__________________<br>(SCT, HBM, SEM, etc)
Barbershop/Hair Salon Recruitment Strategies	__________________
Comparison	__________________<br>(Briefly describe)
Primary Outcome	__________________<br>(Be as specific as possible)
Results	__________________
Significant?	◯ Yes<br>◯ No

Secondary Outcome(s)	__________________
Significant Results	__________________
Feasibility Outcomes	☐ Satisfaction with intervention<br>☐ Intention to continue intervention<br>☐ Practicality<br>☐ Integration<br>☐ Limited efficacy<br>☐ Other<br>☐ Not reported/not applicable<br>(select all that apply)
Other	__________________
Results	__________________
Intervention training	__________________
Measures of Fidelity	__________________
Major limitations	__________________
References to obtain	__________________<br>(additional references to screen)
Notes	__________________
Article PDF	
Extracted By:	◯ Kelly<br>◯ Patrick
Extraction completed on:	__________________
Reviewed by Forest?	◯ Yes<br>◯ No

Reviewed on: ________________________________
