# Supplementary material for: Health promotion interventions for African Americans delivered in U.S. barbershops and hair salons- a systematic review
Source: BMC Public Health. 2021 Aug 16;21:1553. doi: 10.1186/s12889-021-11584-0 (PMC8365990; doi:10.1186/s12889-021-11584-0)
Supplement: Supplementary file 3 — Additional file 3. Quality assessment form. [file 12889_2021_11584_MOESM3_ESM.pdf]

Quality Assessment

Article ID

____
(enter ID # from Covidence)

Selection Bias

Are the individuals selected to participate in the study likely to be representative of the target population?
- ◯ Very likely
- ◯ Somewhat likely
- ◯ Not likely
- ◯ Can't tell

What percentage of selected individuals agreed to participate?
- ◯ 80-100% agreement
- ◯ 60-79% agreement
- ◯ less than 60% agreement
- ◯ Not applicable
- ◯ Can't tell

Rate this section
- ◯ Strong ◯ Moderate
- ◯ Weak

Study Design

Indicate the study design
- ◯ Randomized controlled trial
- ◯ Controlled clinical trial
- ◯ Cohort analytic (two group pre + post)
- ◯ Case-control
- ◯ Cohort (one group pre + post (before and after))
- ◯ Interrupted time series
- ◯ Other
- ◯ Can't tell

Was the study described as randomized?
- ◯ Yes
- ◯ No

Was the method of randomization described?
- ◯ Yes
- ◯ No

Was the method appropriate?
- ◯ Yes
- ◯ No

Rate this section
- ◯ Strong ◯ Moderate
- ◯ Weak

Confounders

Were there important differences between groups prior to the intervention?
- ◯ Yes
- ◯ No
- ◯ Can't tell

(race, sex, martial status/family, age, SES, education, health status, Pre-intervention score on outcome measure)

Indicate the percentage of relevant confounders that were controlled (either in the design (e.g. stratification, matching) or analysis)?	◯ 80 - 100% (most)<br>◯ 60 - 79% (some)<br>◯ Less than 60% (few or none)<br>◯ Can't Tell
Rate this section	◯ Strong ◯ Moderate<br>◯ Weak

Blinding

Was (were) the outcome assessor(s) aware of the intervention or exposure status of participants?	◯ Yes<br>◯ No<br>◯ Can't tell
Were the study participants aware of the research question?	◯ Yes<br>◯ No<br>◯ Can't tell
Rate this section	◯ Strong ◯ Moderate<br>◯ Weak

Data Collection Methods

Were data collection tools shown to be valid?	◯ Yes<br>◯ No<br>◯ Can't tell
Were data collection tools shown to be reliable?	◯ Yes<br>◯ No<br>◯ Can't tell
Rate this section	◯ Strong ◯ Moderate<br>◯ Weak

Withdrawals and Drop-Outs

Were withdrawals and drop-outs reported in terms of numbers and/or reasons per group?	◯ Yes<br>◯ No<br>◯ Can't tell<br>◯ Not applicable (i.e. one time surveys or interviews)
Indicate the percentage of participants completing the study. (If the percentage differs by groups, record the lowest).	◯ 80 - 100%<br>◯ 60 - 79%<br>◯ Less than 60%<br>◯ Can't Tell<br>◯ Not Applicable (i.e. Retrospective case-control)
Rate this section	◯ Strong ◯ Moderate<br>◯ Weak ◯ Not applicable

Intervention Integrity

What percentage of participants received the allocated intervention or exposure of interest?	◯ 80 - 100%<br>◯ 60 - 79%<br>◯ Less than 60%<br>◯ Can't Tell
Was the consistency of the intervention measured?	◯ Yes<br>◯ No<br>◯ Can't tell
Is it likely that subjects received an unintended intervention (contamination or co-intervention) that may influence the results?	◯ Yes<br>◯ No<br>◯ Can't tell

Analyses

Indicate the unit of allocation	◯ Community<br>◯ Organization/Institution<br>◯ Practice/Office<br>◯ Individual
Indicate the unit of analysis	◯ Community<br>◯ Organization/Institution<br>◯ Practice/Office<br>◯ Individual
Are the statistical methods appropriate for the study design?	◯ Yes<br>◯ No<br>◯ Can't tell
Is the analysis performed by intervention allocation status (i.e. intention to treat) rather than the actual intervention received?	◯ Yes<br>◯ No<br>◯ Can't tell

Component Ratings

	Strong	Moderate	Weak
Selection Bias	◯	◯	◯
Study Design	◯	◯	◯
Confounders	◯	◯	◯
Blinding	◯	◯	◯
Data Collection Methods	◯	◯	◯
Withdrawals and Drop-Outs	◯	◯	◯

GLOBAL RATING FOR THIS PAPER	◯ Strong (no weak ratings)<br>◯ Moderate (one weak rating)<br>◯ Weak (2 or more weak ratings)
Is there a discrepancy between the two reviewers with respect to the component ratings?	◯ Yes ◯ No<br>(With both reviewers discussing the ratings)
Indicate the reason for the discrepancy	◯ Oversight<br>◯ Differences in interpretation of criteria<br>◯ Differences in interpretation of study

Final decision of both reviewers	◯ Strong<br>◯ Moderate<br>◯ Weak
